# Suicide by sodium nitrite ingestion: a systematic review

**DOI:** 10.1007/s12024-025-01066-9

**Published:** 2025-08-30

**Authors:** Edoardo Mazzini, Alessandro Feola, Ilaria Fracassi, Anna Carfora, Antonietta Porzio, Santina Alessandra Severino, Mariavictoria De Simone, Carlo Pietro Campobasso

**Affiliations:** 1 Department of Experimental Medicine, University of Campania “Luigi Vanvitelli”, via Luciano Armanni 5, Naples, 80138 Italy; 2 Department of Mental and Physical Health and Preventive Medicine, University of Campania “Luigi Vanvitelli”, via Luciano Armanni 5, Naples, 80138 Italy

**Keywords:** Sodium nitrite, Suicide, Forensic pathology, Post-mortem toxicology

## Abstract

Sodium nitrite (NaNO_2_) is an odorless inorganic compound appearing as a yellowish to white powder, primarily used as a food additive. An alarming increasing trend in suicides involving NaNO_2_ ingestion has been recently observed. The aim of this study is to present a systematic review of intentional NaNO_2_ poisonings, focusing on the epidemiological characteristics of the victims, their psychiatric history, the setting and circumstances of death, as well as autopsy, histological and toxicological results. A total of 24 papers satisfied the inclusion criteria reporting 94 suicides by NaNO_2_ ingestion. Most victims were male (62.8%), belonging to the 13-40-year-old age group. Psychiatric history was available in 43 cases, with depression being the most reported disorder (19 cases). Autopsy and/or external examination were performed in 63 cases, mostly showing unspecific signs of asphyxia and dark discoloration of the hypostasis and blood. Methemoglobin (Met-Hb) levels in blood were available in 49 suicides, ranging from 6 to 92%. In 35 cases levels exceeded 30%, with 19 victims showing concentrations above 50%. Nitrites (NO_2_) and nitrates (NO_3_) were analyzed from different biological samples including blood, urine, gastric content, and other tissues. NO_2_ were detected in 44 cases, while NO_3_ in 30 victims. Blood was the most analyzed matrix with NO_2_ and NO_3_ concentrations ranging from 0.03 µg/mL to 372.65 µg/mL and from 0.899 µg/mL to 1,597.9 µg/mL, respectively. In addition, a case of fatal self-poisoning by oral ingestion of NaNO_2_, which came to the author’s attention, is presented.

**Supplementary Information:**

The online version contains supplementary material available at 10.1007/s12024-025-01066-9.

## Introduction

Sodium nitrite (NaNO_2_) is an inorganic white to yellowish crystalline powder commonly used as a food additive [1]. In healthy individuals, physiological blood concentration of nitrites (NO_2_) and nitrates (NO_3_) typically ranges between 0.1 and 0.4 µmol/L and 20 to 40 µmol/L, respectively [2], originating from both endogenous metabolic pathways and dietary intake [3]. Exposure to elevated levels of NaNO2 can lead to the onset of clinical symptoms like nausea, weakness, numbness, shortness of breath, tachycardia, and cyanosis [4]. The estimated lethal dose of sodium nitrite in adults is approximately 2.6 g [5]. However, individual responses may vary, as demonstrated by documented cases of survival following ingestion of higher doses, up to 6 g [6]. Due to growing safety concerns, a recent European legislation [7] has lowered the maximum allowable concentrations of nitrites and nitrates in animal-derived food products to 75 mg/kg, amending the Food Additives Regulation (EC) No 1333/2008 [8]. Sodium nitrite’s toxicity results primarily from its ability to oxidize the ferrous iron (Fe^²⁺^) in hemoglobin to the ferric state (Fe^³⁺^), leading to elevated levels of methemoglobin (Met-Hb) in blood [9]. Normally, Met-Hb accounts for 1–3% of the total circulating hemoglobin. When levels rise above 50%, severe symptoms such as seizures, arrhythmias, acidosis, and coma may occur, with fatal outcomes commonly seen at concentrations exceeding 70% [5, 10]. Nevertheless, rare cases of patient survival with Met-Hb levels as high as 94% have also been documented [11, 12]. An alarming increasing trend in the use of NaNO_2_ as a suicidal mean has recently been reported in literature [13]. More than 720,000 individuals die by suicide every year, making it the third leading cause of death among individuals aged 15–29 [14]. Self-poisoning is one of the most used methods of suicide [15]. In 2021, the National Poison Data System (NPDS) in the United States reported a 30% increase in suspected self-poisoning suicide attempts among individuals aged 10–19 compared to 2019, with an increase of 73% among 10-12-year-olds and 49% among 13-15-year-olds respectively [16]. The Aim of this study is to conduct a systematic review of NaNO_2_ intentional poisoning, in order to identify common characteristics of these fatal intoxications, focusing on the features of the victims (age, gender, psychiatric disorders), environmental settings, circumstances of death, toxicological and autopsy findings.

## Materials and methods

A literature review was conducted in accordance with the PRISMA statement [17], a standardized methodological framework used for systematic reviews and meta-analyses. The keyword “sodium nitrite AND suicide” was searched in all fields of three electronic databases (PubMed, Scopus, and Web of Science) to identify relevant publications available up to July 22nd, 2025, without time restrictions. Two independent authors screened the articles by title and abstract and subsequently assessed the full text for eligibility based on predefined inclusion and exclusion criteria. Articles were selected if they: (1) were published in English; (2) reported at least one case of suicide by NaNO_2_ ingestion, (3) provided sufficient details regarding the victim(s) (e.g., gender, age, medical or psychiatric history), the death scene (indoor or outdoor), and/or autopsy or external examination findings, including toxicological results. Exclusion criteria included: (1) non-English language publications; (2) unavailable full text; (3) accidental NaNO_2_ poisoning; (4) studies based on animal models, (5) articles reporting survival cases of NaNO_2_ poisoning. For duplicate studies, only the one providing more detailed information was included. Two examiners independently provided the initial selection of the articles; the title, the abstract, and the full text of each potentially pertinent study were reviewed. Any disagreement concerning study inclusion was discussed by the two examiners. If no agreement could be reached, conflict was solved through a third co-author as reviewer. Key data were captured from each article including: first author, year of publication, country, number of cases, features of the victim (gender, age, medical history, psychiatric disorders), death scene, autopsy/external examination findings, histological and/or toxicological findings along with concentrations of NaNO_2_ and Met-Hb.

## Results

**Fig. 1:**
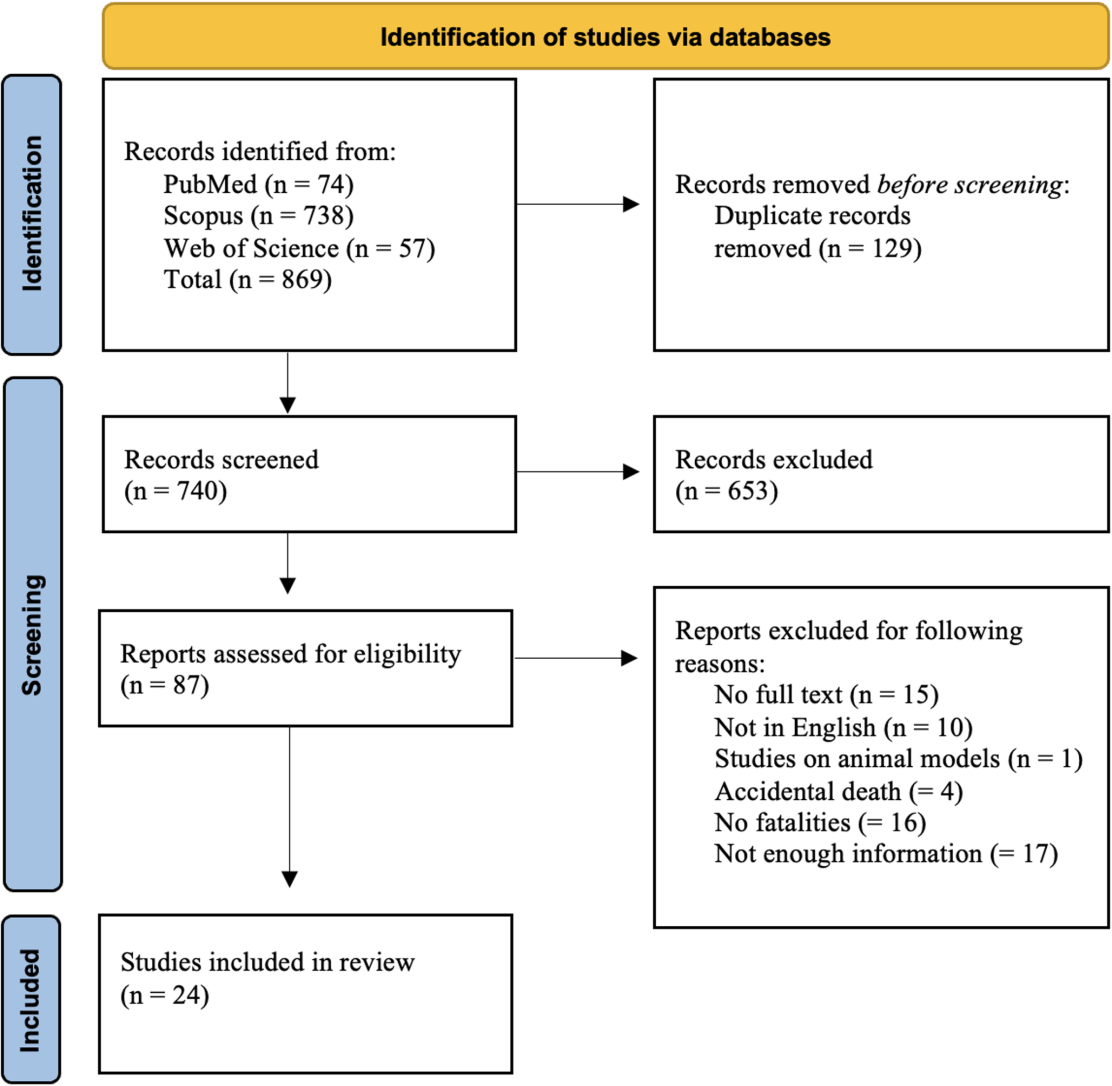
Flowchart depicting the methodology followed in the study

The literature search across PubMed, Scopus, and Web of Science provided a total of 869 articles: 74 from PubMed; 738 from Scopus; and 57 from Web of Science. After removal of 129 duplicates, 740 articles remained. Screening of titles and abstracts led to the exclusion of 653 articles that did not meet the inclusion criteria. A total of 87 articles were identified as potentially relevant and were sought for retrieval. Among these, 15 papers were excluded due to unavailability of the full text, 10 were not published in English, and one involved animal studies. 17 papers were excluded due to insufficient information regarding the victims, the death scene, or post-mortem findings. 16 papers reported non-fatal poisonings, while 4 involved accidental ingestion of NaNO_2_. Ultimately, 24 papers met the inclusion criteria reporting a total of 94 suicides by NaNO_2_ poisoning. A flowchart depicting the selection of studies according to PRISMA standards is reported in Fig. 1. The selected papers report case studies of intentional NaNO_2_ poisoning occurred over a 15-year period from 2010 to 2025 in different countries. Most of the reviewed articles are single case reports. Multiple fatalities up to 5 victims are reported in 6 articles. Three case series reporting 10, 20 and 28 victims respectively have been also selected [1, 18, 19]. Victims of intentional ingestion of NaNO_2_ were mostly from Europe (40 victims reported by 14 papers), followed by Canada with a single case series of 28 victims [19], USA and Oceania, with 13 and 11 victims respectively. Only 2 case reports from Asia were selected [20, 21]. The details of the 94 cases reported by the 24 articles reviewed are summarized in Table 1.

**Table 1:** Characteristics of the victims (age, sex, psychiatric history), setting and circumstances of death, autopsy and histological findings, toxicological findings and analytical methods

Reference	Country	Sex/Age	Psychiatric history	Setting and circumstances of death	Autopsy findings	Histological findings	Met-Hb %	NO2 value	NO_3_ value	Other toxicological findings	Analytical method
***Harvey et al.***, **2010** [41]	New Zealand	M, 76	Depressive syndrome; P.S.A.	E.D.	N.A.	N.A. .	A.M. 82.6%	NaNO2 in GC	N.A.	N.A.	N.A.
***Mudan et al.***, **2020** [24]	U.S.A.	M, 27	N.A.	E.D.	N.A.	N.A.	A.M. >32.4%	N.A.	N.A.	N.A.	N.A.
		F, 16	N.A.	E.D.; NaNO2 purchased online	N.A.	N.A.	A.M. >30%	N.A.	N.A.	N.A.	N.A.
		M, 25	N.A.	E.D.	N.A.	N.A.	P.M. 29%	N.A.	N.A.	N.A.	N.A.
**Durao et al.**,** 2020** [26]	Portugal	M, 37	Depressive syndrome; Schizophrenia; P.S.A.	Outdoor (car); Suicide kit ordered on the internet	**Hypostasis**: brown, grey, blue, red **Cyanosis**: extremities **Other findings**: general signs of asphyxia	Pulmonary edema; Coronary artery disease	N.A.	GC 16,000 mcg/mL GC 24,000 mcg/mL NaNO2 B 0.03 mcg/mL	N.A.	Citalopram; Trazodone	Spectrophotometry for NaNO2
***Neth et al.***., **2021** [25]	U.S.A.	F, 17	Bipolar disorder; substance use disorder; P.S.A.	On route to the E.D.; NaNO2 purchased online	N.P.	N.P.	A.M. 48%	N.A.	N.A.	N.A.	N.A.
***Dean et al.***, **2021** [34]	U.S.A.	F, 24	No history of mental issues	Indoor	**Hypostasis**: Mottled purple-grey **Other findings**: deep red-purple blood with no clots	N.A.	P.M. 62%	N.A.	N.A.	N.D.	Spectrophotometry for Met-Hb
		M, 22	No history of mental issues	Indoor; Suicide notes	**Hypostasis**: blue-grey discoloration of the skin and hand nails **Dark discolouration and increased fluidity of the blood**	Linear hemorrhages in the stomach and intestine mucosa	P.M. 33%	VH 3402,37 mcg/mL Na	N.A.	N.D.	Spectrophotometry for Met-Hb
		M, 39	Personality Disorder; P.S.A.	Indoor	**Hypostasis**: red-purple and grey **Cyanosis**: hand nails **Dark discolouration and increased fluidity of the blood**	hemorrhagic gastritis; Visceral congestion	P.M. 44%	N.D.	N.A.	N.D.	Spectrophotometry for Met-Hb
***Durao et al.***, **2021** [38]	Portugal	F, 37	No history of mental issues	Indoor (bedroom); Suicide note	**Hypostasis**: greyish **Other findings**: general signs of asphyxia	Myocardial changes; Pulmonary edema	N.A.	HB 35mcg/mL	N.A.	Diazepam; Codeine; Quetiapine; Tramadol	Griess method
***Barranco et al.***., **2021** [29]	Italy	M, 31	Depressive syndrome	Outdoor (car)	**Hypostasis**: blue-red **Cyanosis**: hands and feet	Pulmonary edema and intra-alveolar hemorrhages; Myocardial changes	P.M. 73%	N.A.	N.A.	N.A.	UPLC-MS/MS (Ultrahigh-performance liquid chromatography–tandem mass spectrometry technique)
***Hwang et al.***., **2021** [20]	Korea	M, 28	N.A.	Indoor (Home); Logged-in on suicide forum	**Hypostasis**: reddish-purple **Cyanosis**: hands and feet **Petechiae**: hand nails **Other findings**: dark brown color of the face	Visceral congestion; Pulmonary edema	P.M. 33%	GC 11220.1 mcg/mL PF 181.0 mcg/mL	CSF 50.5 mcg/mL GC 137.8 mcg/mL PB 220.0 mcg/mL HB 218.5 mcg/mL PF 91.7 mcg/mL	N.A.	Blood gas analysis (Met-Hb) Ion chromatography (nitrites/nitrates/ sodium nitrite)
***Hickey et al.***, **2021** [19]	Canada	21 M; 7 F 32.8 (SD ± 18.84) aa Min. 17 Max. 86	N.A.	N.A.	**Hypostasis** N.A. (1 case); Unremarkable (7 cases); Different shades of purple (11 cases) Different shades of grey (8 cases) **Dark discolouration of the blood** (1 case)	N.A.	P.M. Met-Hb range 6%-92% Test failed (2 cases) N.A. (3 cases)	N.A.	N.A.	N.A. (3 cases) Negative (9 cases) Antihistamines (10 cases) Antidepressants (6 cases) Antiemetics (1 case) Sub. of abuse (2 cases) Others (4 cases)	N.A.
***Huntington et al.***, **2021** [39]	U.K.	F, 28	N.A.	E.D.	N.A.	N.A.	A.M. 81%	N.A.	N.A.	N.A.	N.A.
***Tomsia et al.***, **2021** [31]	Poland	M, 23	N.A.	Indoor ( bathroom)	**Hypostasis**: dark purple discoloration of the lips **Cyanosis**: hands and fingernails	N.A.	N.D.	B 0.2 mcg/mL U 24.6 mcg/mL GC 220 mcg/mL VH 57.7 mcg/mL Costal cartilage 3.4 mcg/mL Kidney 3.6 mcg/mL	N.A.	Negative	Griess method
***Taus et al.***, **2021** [33]	Italy	M, 28	Depressive syndrome (mild)	Indoor	**Hypostasis**: brown-red **Cyanosis**: Lips; finger nails	Pulmonary edema; Visceral congestion	N.A:	traces on blood	B 403.031 mcg/mL	PB Et-OH	Capillary electrophoresis operating in the CIA mode coupled with UV detection
		M, 33	Depressive syndrome (severe)	Indoor (Living room)	**Hypostasis**: grey-blue-red **Cyanosis**: extremities	N.A.	N.A.	N.D.	B 272.82 mcg/mL	Delta-THC	+
***Bugelli et al.***, **2022** [30]	Italy	M, 51	No history of mental issues	Indoor (Bedroom); Suicide note	**Hypostasis**: greyish **Cyanosis**: Hands (Subungueal) **Dark discolouration of the blood** **Other findings**: pulmonary edema; visceral congestion	Myocardial changes; Pulmonary edema and emphysema	P.M. >30%	N.D	PB 460 mcg/mL U 48.3 mcg/mL	Alprazolam; Et-OH	HPLC coupled to High-Resolution Mass Spectrometry (HRMS); LC-MS (Blood gas analysis using GEM Premier 5000 (Werfen) to detect Met-Hb)
		M, 35	Anxious-depressive syndrome	Indoor (Bathroom)	**Hypostasis**: greyish and purple **Cyanosis**: Hand nails **Dark discolouration of the blood** **Other findings**: pulmonary edema; visceral congestion	Myocardial changes; Pulmonary emphysema and intra-alveolar haemorrhages	N.A.	N.D.	PB 280 mcg/mL U 269 mcg/mL	Mirtazapine	+
		F, 44	No history of mental issues	Indoor; Suicide note	**Hypostasis**: purple-bluish **Cyanosis**: Lips and hand nails	Visceral congestion; pulmonary emphysema; brain edema; Myocardial changes	P.M. >30%	N.D.	PB 378 mcg/mL U <10 mcg/mL	Metoclopramide	+
		M, 27	No history of mental issues	Outdoor (mountainous area); Called suicide hotline declaring he was going to commit suicide	**Hypostasis**: blue-greyish **Cyanosis**: Lips and hand nails	Visceral congestion; Pulmonary emphysema	P.M. >30%	N.D.	PB 311 mcg/mL U 102 mcg/mL	Metoclopramide	+
***Wettstein et al.***, **2022** [36]	U.S.A.	M, 25	No history of mental issues	E.D.	**Other findings**: brown discoloration of the muscle tissue	N.A.	A.M. 28.8%	B Nitrate + Nitrite 5300 micromol/L	B Nitrate + Nitrite 5300 micromol/L	N.A.	N.A.
***Mun et al.***, **2022** [21]	Korea	M, 26	N.A.	E.D.	N.A.	N.A.	A.M. 90.3%	N.A.	N.A.	N.A.	N.A.
***Stephenson et al.***, **2022** [18]	Australia	M, 74	Depressive syndrome; P.S.A.	N.A.; Informed friends about his intentions to commit suicide	**Hypostasis**: blue-grey **Dark discolouration of the blood** **Other findings**: pulmonary edema	N.A.	N.A.	N.A.	N.A.	Diazepam; Venlafaxine Amlodipine; Paracetamol; ​​Prochlorperazine	N.A.
		M, 60	Depressive syndrome	N.A.; Suicide note	**Hypostasis**: blue-grey **Dark discolouration of the blood** **Other findings**: pulmonary edema	N.A.	P.M. 87.5% CO-Hb 31%	N.A.	N.A.	Venlafaxine; Desvenlafaxine; Paracetamol; Buprenorphine; Metformin; Mirtazapine	N.A.
		M, 74	Anxious-depressive syndrome;	N.A.; Suicide note	**Hypostasis**: blue-grey **Dark discolouration of the blood** **Other findings**: pulmonary edema	N.A.	CO-Hb 51% Met-Hb outside analytical limit	Urine dipstick positive	N.A.	Lorazepam; Fentanyl; Metoclopramide; Pregabalin	N.A.
		M, 69	Depressive syndrome	N.A.; Suicide note	**Hypostasis**: blue-grey **Dark discolouration of the blood** **Other findings**: pulmonary congestion	N.A.	Sample not suitable	Urine dipstick positive	N.A.	Sertraline	N.A.
		F, 29	Depressive syndrome; P.S.A.	N.A.; Farewell messages on the phone; Suicide note	**Hypostasis**: blue-grey **Dark discolouration of the blood** **Other findings**: pulmonary congestion and edema	N.A.	CO-Hb 25%	Urine dipstick positive	N.A.	Naloxone	N.A.
		M, 64	No history of mental issues	N.A.	**Hypostasis**: blue-grey **Dark discolouration of the blood** **Other findings**: pulmonary edema	N.A.	Met-Hb N.D.	Urine dipstick positive	N.A.	Paracetamol; Codeine; Prochlorperazine; Ranitidine; Loperamide	N.A.
		F, 23	Personality disorder;	N.A.; Suicide note; Internet search about suicide methods	**Hypostasis**: blue-grey **Dark discolouration of the blood** **Other findings**: pulmonary edema and congestion	N.A.	CO-Hb 51% Met-Hb N.D.	Urine dipstick positive	N.A.	Metoclopramide; Quetiapine; Paracetamol	N.A.
		M, 64	Depressive syndrome	N.A.; Suicide notes	**Hypostasis**: blue-grey **Dark discolouration of the blood** **Other findings**: pulmonary edema and congestion	N.A.	CO-Hb 40% Met-Hb N.D. AM	Urine dipstick positive	N.A.	Et-OH; Metoclopramide; Paracetamol; Zolpidem	N.A.
		M, 22	N.A.	N.A.; Suicide notes	**Hypostasis**: blue-grey **Dark discolouration of the blood**	N.A.	Met-Hb N.D.	Urine dipstick positive	N.A.	Metoclopramide; Ranitidine; Paracetamol	N.A.
		M, 40	N.A.	N.A.; Suicide notes	**Other findings**: Advanced putrefactive changes	N.A.	Met-Hb N.D.	Urine dipstick positive	N.A.	Paracetamol; Metoclopramide; Et-OH	N.A.
***Loiseau et al.***, **2023** [22]	France	F, 33	Spectrum autism disorder	Indoor (Bedroom)	**Hypostasis**: purplish **Cyanosis**: Lips and ears **Petechiae**: inner surface of the scalp **Other findings**: Visceral congestion; whitish foam in the tracheobronchial tree	Visceral congestion; intra-alveolar hemorrhages	N.A.	GC 30.9 mcg/mL	N.A.	Metoclopramide	RAMAN spectrometry; Spectrophotometric study using the Saltzman’s reagent for Gastrc Content analysis
***Szórádová et al.***, **2023** [28]	Slovak Republic	F, 19	No history of mental issues	Outdoor (Vehicle)	**Hypostasis**: grey-purple; grey-brown **Petechiae**: subpleural effusions **Dark discolouration and increased fluidity of the blood** **Other findings**: Visceral congestion	N.A.	P.M. >70%	N.A.	N.A.	N.A.	N.A.
		M, 24	Personality disorder; P.S.A.	Outdoor (Cornfield)	**Hypostasis**: grey-blue; grey-brown **Dark discolouration and increased fluidity of the blood** **Other findings**: Visceral congestion	N.A.	P.M. 20.24%	Detected in GC and U, but value not specified	Detected in B, GC and U, but value not specified	Acetylsalicylic acid; Ibuprofen metabolite	Isotachophoresis with a conductivity detector.
		F, 33	Depressive syndrome	Indoor (Home)	**Hypostasis**: grey-blue-purple **Petechiae**: subpleural effusions **Dark discolouration and increased fluidity of the blood** **Other findings**: Visceral congestion	N.A.	P.M. 54.5%	5.51 mcg/mL	219,72 mcg/mL	Metoclopramide	N.A.
		M, 21	Personality disorder	On route to the E.D.	**Hypostasis**: grey-purple **Petechiae**: subpleural effusions **Dark discolouration and increased fluidity of the blood** **Other findings**: Old scars on both forearms; Visceral congestion	N.A.	P.M. 71.4%	N.A.	N.A.	N.A.	N.A.
***Andelhofs et al.***, **2023** [32]	Belgium	F, 18	Mental health issues; P.S.A.	Indoor (Bedroom)	**Hypostasis**: grey-brownish **Cyanosis**: Lips and fingernails **Other findings**: Healing self-harm injuries on the forearms	Pulmonary edema and intra-alveolar hemorrhages; Visceral congestion; Liver steatosis	P.M. 35%	Serum 38 mcg/mL GC 1150 mcg/mL	N.A.	Negative	Spectrophotometric analysis; Griess reaction; Macherey–Nagel.
***Hikin et al.***, **2023** [1]	U.K.	F 8; M 12 31.3 (SD ± 11.5) Min. 14 Max. 49	P.S.A (10 cases) Depressive syndrome (4 cases) Personality disorders (2 cases) Other mental health issues (5 cases) Eating disorder (1 case) N.A. (4 cases) Cases with >1 condition (1)	Indoor (Bedroom 6 cases, Home 1 case, Living room 2 cases) on route to E.D. (1 case) E.D. (1 case) N.A. (9 cases) Suicide pact (2 cases)	N.A.	N.A.	N.A.	Blood range 0.107–372.64 mcg/mL B ND (1 case)	Blood range 0.899mcg/mL–1308 mcg/mL B traces (1 case) B N.D. (1 case)	Et-OH or illicit drugs (13 cases) Paracetamol (9 cases) Antiemetics (6 cases) Antidepressants (4 cases) Antihistamines (2 cases) Antipsychotics (1 case) Anticonvulsants (1 case) N.D. (2 cases) Others (4 cases)	Gas-phase chemiluminescence
***Zhang et al.***, **2023** [23]	U.S.A.	F, 19	Depressive syndrome; post-traumatic stress disorder; ADHD; Borderline personality disorder	Indoor (Bedroom)	N.A.	N.A.	Sample unsuitable	U negative Serum positive VH positive	N.A:	N.A.	Mquant Nitrite Test Strip with Griess method
		F, 43	Anxious-depressive syndrome; P.S.A.	Indoor (Hotel room)	N.A.	N.A.	Sample unsuitable	U negative VH positive	N.A.	N.A.	Mquant Nitrite Test Strip with Griess method
		F, 61	Depressive syndrome (not diagnosed)	Indoor (Hotel room)	N.A.	N.A.	Sample unsuitable	U positive VH positive	N.A.	N.A.	Mquant Nitrite Test Strip with Griess method
		F, 16	Anxious-depressive syndrome; P.S.A.	E.D.	N.A.	N.A.	A.M. 8.5%	U positive VH positive Serum positive	N.A.	N.A.	Mquant Nitrite Test Strip with Griess method
		F, 41	Suicidal thoughts	On route to the E.D	N.A.	N.A.	A.M. 58%	Serum positive VH positive	N.A.	N.A.	Mquant Nitrite Test Strip with Griess method
***Zerbo et al.***., **2023** [27]	Italy	F, 20	Eating disorder (Anorexia)	Indoor (Bedroom); Handwritten note on how to consume NaNO2 along with the website	**Hypostasis**: greyish-purple **Cyanosis**: Lips **Petechiae**: subpleural; laryngeal; glottal; tracheal mucosa **Other findings**: foamy liquid in the tracheal lumen and at lung section	N.P.	P.M. 12.8%	N.A.	N.A.	Negative	AVOXimeter 4000 ITC oximeter on central blood to detect Met-Hb
***Ječmenica et al.;*** **2024** [35]	Serbia	F, 47	Depressive syndrome	Indoor (Home)	**Hypostasis**: grey-brownish; **Other findings**: foamy liquid in the tracheal lumen; lung congestion	N.P.	P.M. 60.3%	PB 159.8 mcg/mL	PB 1597.9 mcg/mL	Metoclopramide; Risperidone	Griess spectrophotometry (NO_2_) UV spectrophotometry (NO_3_) Evelyn-Malloy’s spectrophotometry (Met-Hb)
***Kaubrytė et al.;*** **2025** [40]	Lithuania	F, 19	N.A.	Indoor (Home); Suicide note	**Hypostasis**: cherry-brownish; **Petechiae**: brain **Other findings**: visceral congestion; pulmonary edema with foamy liquid; **Dark discolouration of the blood**	N.P.	N.P.	GC positive	N.P.	Et-OH; Amphetamines; Atropine; Quetiapine	N.A.

Notes: P.S.A. Previous suicide attempt; HB (Heart blood); PB (Peripheral blood); U (Urine); VH (Vitreous Humor); GC (Gastric content); PF (Pericardial fluid); CSF (Cerebrospinal fluid); N.D. (Not detectable); N.A. (Not available); N.P. (Not performed)

### Gender differences and age groups

Most victims were male (59 out of 94; 62.8%). The age ranged from 13 to 86 years, with a mean value of 44.4 years. Females were younger than males, with a mean age of 29.4 years compared to the latest (mean age of 36.2 years). Most of the victims belonged to the 13–40-year-old age group (68 out of 94; 72.3%), 55 of which were younger than 30 years old. 7 victims were minors [1, 19, 22, 23, 24, 25]. 12 victims were older than 50 years old and exclusively male, except for one 61-year-old woman [23]. The oldest victim was an 86-year-old man with unknown psychiatric history [19].

### Psychiatric disorders

Psychiatric medical records were available for 43 out of 94 victims (45.7%), with no significant gender differences observed within the study sample. Among those with documented psychiatric disorders, 23 were male and 20 were female. No medical history was available in 42 cases, while 9 victims had no psychiatric diagnosis prior to death. Four victims presented with more than one psychiatric disorder [1, 23, 25, 26]; all were under 20 years of age, except for a 37-year-old woman [26] diagnosed with both depressive syndrome and schizophrenia, and with a history of suicide attempts. Depressive syndrome was the most frequently diagnosed condition, observed in 19 out of 43 cases (44.2%), followed by unspecified mental illnesses (6 cases; 13.9%) and personality disorders (5 cases; 11.6%). Two female victims, aged 20 and 49 respectively, suffered from eating disorders [1, 27]. Previous suicide attempts were documented in 20 cases, including 5 individuals with no formal psychiatric diagnosis. Of the 10 victims with depressive syndrome and prior suicide attempts, only one, a 13-year-old girl, was diagnosed with autism spectrum disorder and suicidal ideation [22].

The characteristics of the 94 victims (gender, age, medical history, psychiatric disorders) are summarized in Table 2.

**Table 2:** Distribution of age, gender, and psychiatric disorders across the study sample

Characteristics of the victims	No. of victims = 94
**Gender**	
Female Male	35 (37.2%) 59 (62.8%)
Age <20 years 21–30 years 31–40 years 41–50 years 51–70 years >70 years	[Mean age = 44.4] 19 (20.2%) 36 (38.3%) 13 (13.9%) 14 (14.9%) 7 (7.4%) 5 (5.3%)
Psychiatric disorders Depressive syndrome Personality disorders Mental health issues Autism spectrum disorders Substance dependence Eating disorders More than one psychiatric diagnosis P.S.A. with no psychiatric diagnosis P.S.A. with psychiatric diagnosis No psychiatric history reported Not available	43 19 (44.2%) 5 (11.6%) 6 (14.0%) 1 (2.3%) 1 (2.3%) 2 (4.6%) 4 (9.4%) 5 (11.6%) 15 (34.9%) 9 42

### Setting and circumstances of death

Information regarding the location of the suicidal act was available in 34 cases. Most deaths occurred in enclosed settings (29 out of 94; 30.9%), with 27 victims found at home (87.1%). Specifically, 12 individuals were found in their bedroom (38.7%); 3 in the living room, including two men aged 27 and 38 who were found deceased on a sofa after reportedly entering into a “suicide pact” (mutual agreement between two or more people to end their lives together, usually at the same time and by the same method) (9.8%), and two in the bathroom (6.4%). Details about the specific location within the house were not provided in 10 cases (32,3%). 2 victims were found in hotel rooms [23], while three others (two males and one female) were found inside their vehicles [26, 28, 29]. Only two victims died outdoors in public areas: a 24-year-old man found in a cornfield [28]; and a 27-year-old man who contacted a suicide hotline from a mountainous area to declare his suicidal intentions [30]. 13 victims out of 94 (13.9%) died on route to the hospital or shortly after arrival in the Emergency Department (E.D.). In 13 cases, handwritten suicide notes or farewell messages sent via mobile phone to relatives or friends were detected. Sodium nitrite bags and/or suicide paraphernalia were found close to the bodies in all cases; however, online purchase was confirmed in 8 cases only. According to the investigation reports, only two victims did on-line research about NaNO_2_ [18, 30]. One 28-year-old man was found logged-in and chatting about suicide [14]. In another case, next to the body of a 20-year-old woman, a handwritten note with specific instructions on how to consume NaNO_2_ was found [27]. Compared to the whole sample, the victims who had purchased NaNO_2_ online were younger, ranging from 16 to 40 years of age, with a mean age of 26.8 years.

### Autopsy and histological findings

An autopsy and/or external examination was performed in 63 cases. Among these, hypostasis was documented in 51 cases (81%), with discoloration described in different shades of red, purple, brownish, or blue in 41 victims, and greyish in 10 cases. In 7 cases lividity was reported as unremarkable. Cyanosis was observed in 14 victims, mainly localized to the fingernails (7 cases), lips (6 cases); hands and feet (4 cases) and ears in only one case. Petechial hemorrhages were identified in 7 cases: four involved the subpleural region, one of which was associated with petechiae on the laryngeal, glottal, and tracheal mucosa, while additional sites included the fingernails, the inner surface of the scalp, and brain parenchyma. General signs of asphyxia were noted in two cases. Visceral congestion was observed in 14 victims and pulmonary edema in 11. A dark discoloration of the blood and/or an increased fluidity was reported in 19 cases. Moreover, 3 victims showed the presence of whitish foam in the upper and lower airways. Histological investigation was reported for 13 cases only. Of these, pulmonary edema was confirmed in 7, while intra alveolar hemorrhages and pulmonary emphysema were reported in 4 and 3 victims, respectively. Additionally, myocardial alterations and coronary artery disease were documented in four cases. Brain and liver edema were each observed in a single case. General visceral congestion was also observed in 6 victims.

Post-mortem findings reported at autopsy and/or external examination across the study sample are summarized in Table 3.

**Table 3:** Distribution of post-mortem findings across the study sample

Post-mortem findings	
**Hypostasis**	
Red, purple, blue, brownish Greyish/grey Unremarkable	41 10 7
**Cyanotic changes**	
Fingernails Lips Extremities Ears	7 6 4 1
**Petechial hemorrhages**	
Subpleural Laryngeal, glottal, and tracheal mucosa Fingernails Inner surface of the scalp Brain	4 1 1 1 1
Unspecific signs of asphyxia	2
Visceral congestion	14
Pulmonary edema	11
Darker color and increased fluidity of the blood	19
White foam in tracheobronchial tree	3

### Post-mortem and ante-mortem toxicology

#### Methemoglobinemia (Met-Hb)

Met-Hb levels were available in 49 cases (52.1%), with 40 victims tested after death and 9 upon arrival at the emergency department (E.D.). Met-Hb levels provided by the pre-mortem toxicology ranged from 8,5% [23] to 90,3% [20], with a mean value of 42,67%. Among them, 2 victims showed Met-Hb levels below 30%, while 7 exceeded 30%, including 4 with concentrations greater than 50%. Post-mortem Meth-Hb levels, determined from blood samples collected during autopsy, ranged from 6% [19] to 92% [19], with a mean value of 43,4%. Concentrations below 30% were reported in 12 cases, while 28 victims showed levels above 30%, including 15 exceeding 50%. In 5 cases [18], blood concentration of carboxyhemoglobin (CO-Hb) was also measured, providing results from 25% up to 51%, with a mean value of 40%. Met-Hb levels were not assessed in 32 cases, while in 13 cases the analysis didn’t return a valid result due to sample unsuitability. A summary of Met-Hb and CO-Hb findings is presented in Table 4.

**Table 4:** Distribution of Met-Hb and CO-Hb levels across the study sample

Met-Hb	No. of victims = 94	Met-Hb range
**Ante-mortem** < 30% 30–50% >50% **Post-mortem** < 30% 30–50% >50% N/V* N/A*	9 (9.6%) 2 3 4 40 (42.5%) 12 13 15 13 (13.9%). 32 (34.0%)	8.5% - 90.3% [Mean value = 42,67%] 6% − 92% [Mean value = 43,4%] - -
**CO-Hb**	**No. of victims = 5**	25% − 51% [Mean value = 40,0%]

N/A = Not available; N/V = Not valid

### Sodium nitrite, nitrites and nitrates

Toxicological analysis of nitrites (NO_2_) and nitrates (NO_3_) were performed in 45 cases using different methods such as: chemiluminescence in 20 cases (44.4%); spectrophotometry, liquid chromatography and colorimetric essay in 5 cases each (11.1%); Griess Method and capillary Electrophoresis in 2 cases each (4.4%); ion chromatography, spectroscopy, isotachophoresis, co-oximetry, Griess and UV spectrophotometry in one case each (2.2%). Analyzed samples included central blood, peripheral blood, urine, gastric content and vitreous humor. In one case, samples from the costal cartilage and kidneys were also collected [31]. 44 victims tested positive for NO_2_, 24 of which in association with NO_3_. NO_2_ analysis on blood showed positive results in 29 cases ranging from 0.03 µg/mL [26] to 372.65 µg/mL [1]; serum was positive for nitrites in 3 victims [23] and quantified in one victim only (38 µg/mL) [32]. Traces of nitrites on whole blood samples were detected from one victim only [33]. NO_2_ were detected on gastric content samples from 8 victims, providing proper quantification in 5 of them, from 30.9 µg/mL [22] to 16,000 µg/mL [26]. Stephenson et al. tested urine samples with dipsticks, providing positive results in 8 cases whereas the concentration of NO_2_ found by Tomsia et al. [31] was 24.6 µg/mL. Other positive samples included vitreous humor (6 cases); pericardial fluid (1 case), costal cartilage (1 case) and kidney (1 case). Dean et al. [34] tested samples from vitreous humor for sodium in one case, providing a result of 3,402 µg/mL; Durao et al. [26] found NaNO_2_ from gastric content in a concentration of 24,000 µg/mL.

NO_3_ presence was detected in 30 cases, among which 24 in association with NO_2_. Analysis for NO_3_ on blood tested positive in 29 cases, ranging from 0.899 µg/mL to 1,597.9 µg/mL [1, 35]. In one case [20], NO_3_ was tested both on central and peripheral blood, providing results of 218.5 µg/mL and 220 µg/mL, respectively. NO_3_ was also detected from the peripheral blood and urine of four victims, providing results from 280 µg/mL to 460 µg/mL and from less than 10 µg/mL up to 269 µg/mL, respectively [30]. Wettstein et al. [36] detected a 5,300 µmol/L blood concentration of nitrites and nitrates in combination. NO_3_ was also detected on gastric content from 2 victims [20, 28]. In one case, samples from pericardial fluid and cerebrospinal fluid were collected, providing a concentration of 50.5 µg/mL and 91.7 µg/mL [20]. Table 5 summarizes the concentrations of NO_2_ and NO_3_ detected in the study sample.

**Table 5:** Distribution of nitrites and nitrates levels across the study sample

	Blood	Urine	Gastric Content	Others
**Nitrites (44 cases)**	29 cases; Min. 0.03 µg/mL; Max. 372.65 µg/mL	9 cases; Not quantified-24.6 µg/mL	8 cases; Min. 30.9 µg/mL; Max. 16,000 µg/mL	Vitreous humor (6 cases); Pericardial fluid (1 case); Costal cartilage (1 case); Kidney (1 case).
**Nitrates (30 cases)**	29 cases; Min. 0.899 µg/mL; Max. 1,597.9 µg/mL	4 cases; Min. 10 µg/mL; Max. 269 µg/mL	2 cases	Pericardial fluid (1 cases): 50.5 µg/mL; Cerebrospinal fluid (1 case): 91.7 µg/mL

### Alcohol and substances of abuse

Toxicological analyses of licit and illicit drugs were available in 74 out of 94 cases (78.7%), with 31 victims positive for more than one substance. Antidepressants were the most frequently detected drugs (22 out of 74 cases; 29.7%) followed by illicit drugs (20 out of 74; 27%) and antiemetics (20 victims); 14 victims tested positive for antihistamine drugs (14 out of 74; 19%). Of particular relevance is the presence of the last two drug classes, since the so called “suicide kits” available online often include both antiemetic drugs and/or generic antiacids. Their use is also suggested on websites that provide step-by-step instructions on how to commit suicide by NaNO_2_ ingestion. In two cases, a 23-year-old woman and a 74-year-old man, anticonvulsants were detected [1, 18]. None of the detected substances played a role in the determination of death. Among the 72 victims for whom standard toxicological analysis were available, 17 (23.6%) tested negative for ethanol as well as for licit or illicit drugs. Results are summarized in Table 6.

**Table 6:** Results from toxicological analysis conducted on the study sample

Toxicological analysis	No. of victims = 94
Positive Antidepressants Et-OH Antiemetics Paracetamol Antihistamines Others Illicit drugs (opioids / THC / cocaine) Antipsychotics Anticonvulsants	57 (60.6%) 22 7 20 16 14 15 20 5 2
Negative	17 (18.1%)
**N/A**	**20 (21.3%)**

An additional case of fatal intentional NaNO_2_ intoxication came to the author’s attention and it is reported below.

## Case report

**Fig. 2:**
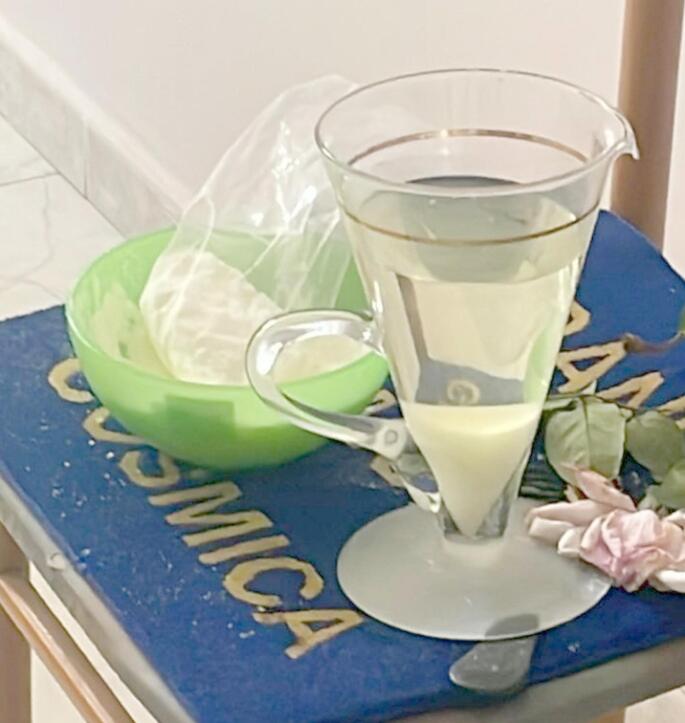
Bowl and glass pitcher found on the death scene

A 30-year-old woman was found unconscious lying on the floor in her bedroom by her mother. The bedroom’s floor was covered with liquid yellow-greenish vomit. During the inspection, a bowl containing a large amount of white-yellowish crystal-powder was found along with a cone-shaped glass pitcher containing about 1 L of a yellowish liquid substance with some crystal powder precipitated on the bottom (Fig. 2). Samples from the vomit on the floor, the liquid substance in the pitcher and the crystal powder in the bowl were collected for toxicological analysis. According to her medical records, the victim suffered from unspecified psychiatric issues. She had been hospitalized after a psychotic episode with auditory and visual hallucinations. No prior suicide attempts were reported but, according to a victim’s friend, the decedent had more than once declared that she no longer wanted to live and her intention to commit suicide by bleach ingestion. At external examination, there was no evidence of self-harm injuries. Lividity showed dark-brownish hypostases consistent with the supine position of the body at the death scene. Cyanosis of the fingernails, toenails and lips was also observed, with the oral mucosa being dotted with bright red petechial hemorrhages. At the autopsy, internal organs were markedly congested with a dark brownish hue of the blood. The stomach contained around 50 ml of dark greenish material of liquid consistency that was collected for toxicological analysis. Samples of blood (cardiac and peripheral) and urine were also collected for toxicological analysis. In histology, a multi-visceral congestion was found. Analysis of the inorganic anions from biological samples were performed with Gas Chromatography coupled to mass Spectrometry (GC/MS), using Pentafluorobenzyl bromide (PFB-Br) as derivatization agent. Extractive alkylation was conducted for 30 min at 60 °C [37]. Analysis revealed the presence of NO_2_ in blood (8.7 µg/ml), urine (0.05 µg/ml) and gastric content (146.6 µg/ml). The concentration of Met-Hb in blood was 59%. Toxicological investigation was also performed on vomit samples collected from the floor during inspection, detecting NO_2_ at a concentration of 20.1 µg/ml. The toxicological analysis was negative for alcohol and licit/illicit drugs.

## Discussion

Results from this systematic review highlight the severity of a phenomenon that has shown a disturbing rise in recent years: the ingestion of NaNO_2_ for suicidal intention [26, 30, 34, 38]. From 2010 to 2025, a total of 94 cases has been reported worldwide, including several European countries [26, 35, 38, 39, 40], Oceania [18, 41], USA [24], Canada [19] and Asia [20]. A comparable upward trend has been also observed in Italy, with seven suicide cases reported since 2022 from different geographical areas of the country [27, 29, 30]. Victims ranged in age from 13 to 86 years (mean value 44.4), with 55 of them under the age of 30 and 7 younger than 18 years old. Psychiatric disorders were documented for 43 victims (45.7%), mainly represented by depressive syndrome and personality disorders. Two female victims had a history of eating disorders, and a 13-year-old girl suffered from autism and depression. In 20 cases, previous suicide attempts were also reported. The features of the victims show how the phenomenon of suicide by NaNO_2_ ingestion predominantly affects a vulnerable population of young individuals with mental health disorders. The profile of the victim in our case is consistent with patterns described in literature: a 30-year-old young woman suffering from mistreated psychiatric disorders and suicidal ideation. In all 94 cases reviewed in this systematic analysis, including our case, NaNO_2_ bags and/or suicide-related paraphernalia were found near the victim’s body. However, online purchase was confirmed in 8 cases only. Two victims had conducted online searches about NaNO_2_ [18, 30] and a 28-year-old man was found logged into an online chat discussing about suicide [20]. Additionally, a handwritten note containing specific instructions on how to consume NaNO_2_ along with the related website was found next to the body of a 20-year-old woman in one case [27]. The online promotion of suicide methods through social media platforms and dedicated websites has emerged as a serious public health concern. In recent years, several pro-suicide forums have gained popularity by promoting NaNO_2_ as a “peaceful” mean of suicide, highlighting its mechanism of action and even offering so-called “suicide kits” containing nitrites, antiemetic medications such as metoclopramide, and detailed, step-by-step instructions readily accessible to users [42, 43]. In 2021, the Prosecutor’s Office in Rome ordered the shutdown of an international online forum with more than 17,000 members actively exchanging information about suicide [44]. The growing international concern over the online availability of sodium nitrite, particularly to minors, has been further emphasized by high-profile legal cases, such as those involving Kenneth Law in Canada and Leonid Zakutenko in the UK; both accused of supplying NaNO_2_ to vulnerable individuals who later committed suicide. Also, the COVID-19 pandemic and the subsequent rise in social media influence may have played a major role in the escalation of this manner of suicide due to isolation, social retreat, anxiety, and depression [18]. An autopsy and/or external examination was performed in 63 cases included in the review. The majority of the victims showed signs of asphyxia due to severe oxygen deprivation, mainly represented by cyanosis of the extremities, petechial hemorrhages of the upper airways, pulmonary edema and visceral congestion. Notably, dark discoloration of the lividity was observed in most of the victims (51), while dark discoloration of the blood was reported in 19 cases. Such signs should alert pathologists to assess the concentration of Met-Hb, as they reflect the oxidation of the ferrous iron to ferric iron in hemoglobin. Met-Hb levels were available in 49 out of 94 cases (52.1%), with 40 measured post-mortem and 9 victims tested upon the arrival at the E.D. Blood samples analysis, conducted both ante-mortem and post-mortem, revealed Met-Hb concentrations ranging from 6 to 92%, with levels exceeding 50% in 19 out of 49 victims. It is widely recognized in literature that Met-Hb blood levels above 70% are frequently fatal [5, 10]. However, the significant variability in Met-Hb levels observed among victims of NaNO_2_ poisoning suggests that death may also result from different mechanisms of cardiovascular toxicity associated with nitrite ingestion, as proposed by some authors [32]. Additionally, the potential post-mortem decrease in Met-Hb blood concentrations should be taken into account when conducting toxicological analysis [20, 45]. NO_2_ and NO_3_ have been also analyzed from different biological samples including blood, urine, gastric content, and other tissues. NO_2_ were detected in 44 cases, while NO_3_ in 30 victims. Blood was the most analyzed matrix with NO_2_ and NO_3_ concentrations ranging from 0.03 µg/mL to 372.65 µg/mL and from 0.899 µg/mL to 1,597.9 µg/mL, respectively. According to toxicological data reported by several authors, NO_3_ levels could, in some cases, significantly exceed NO_2_ levels, highlighting the importance of routinely quantifying both in suspected NaNO_2_ intoxication cases [26, 46]. In our case report, only NO_2_ concentration was analyzed in blood (8.7 µg/ml), urine (0.05 µg/ml) and gastric content (146.6 µg/ml). A Met-Hb concentration of 59% was also found. Among the 94 cases, toxicological analyses were available in 74 (78.7%). 22 victims (29.7%%) tested positive for antidepressants, followed by illicit drugs in 20 (27%). Anticonvulsants were found in two victims, while antiemetics (*n* = 20) and antihistamines (*n* = 14) in a total of 34 cases. The ingestion of the last two drug classes has been documented by several authors [1, 32], as their use is often recommended in online suicide forums as a precautionary measure before the ingestion of NaNO_2_. Data from the systematic review revealed considerable heterogeneity in the measurement of NO_2_, NO_3_ and Met-Hb concentration, suggesting that pathologists frequently rely on circumstantial data to establish the cause of death. While the importance of a thorough crime scene investigation in determining the cause and manner of death is well recognized, a full panel of toxicological analysis, including Met-Hb and NO_2_/NO_3_ concentration in biological samples, should be always conducted in cases where NaNO_2_ poisoning is suspected.

## Conclusions

Although deaths due to NaNO_2_ poisoning remain relatively rare, they represent a significant challenge for medical examiners. The unregulated availability of this compound, combined with the widespread dissemination of detailed instructions on how consuming it through online suicide forums, justifies concerns about a potential rise in such suicides. It is crucial to face this alarming issue, especially among vulnerable young individuals experiencing suicidal ideation. The forensic investigation of NaNO_2_ poisoning-related suicides highlights the need for a comprehensive and multidisciplinary approach. Determining the cause and manner of death requires not only toxicological analyses, but also a meticulous assessment of victims’ historical background, psychological profile, crime scene evidence, and post-mortem findings. A systematic and coordinated investigation process is essential to gain a deeper understanding of these events and to offer insights for prevention strategies.

### Key points

- Sodium nitrite is an odorless inorganic compound mainly used as a food additive.
- Ingestion of sodium nitrite causes an increase of Methemoglobin blood concentration, leading to clinical symptoms related to cellular hypoxia.
- An alarming increasing trend in suicides by NaNO_2_ ingestion has been observed in the last few years.
- Post-mortem findings mainly include dark discoloration of the blood and unspecific signs of asphyxia.
- A full panel of toxicological analysis, including Met-Hb and NO_2_/NO_3_ levels from biological samples, should always be performed in cases where NaNO_2_ poisoning is suspected.

## Supplementary Information

Below is the link to the electronic supplementary material.

Supplementary Material 1

## Data Availability

The data presented in this study are available in request from the corresponding author. The data are not publicly available due to privacy restrictions.
