# Supplementary material for: Suicide by sodium nitrite ingestion: a systematic review
Source: Forensic Sci Med Pathol. 2025 Aug 30;22(1):404–25. doi: 10.1007/s12024-025-01066-9 (PMC13132881; doi:10.1007/s12024-025-01066-9)
Supplement: Supplementary file 1 — Supplementary Material 1 [file 12024_2025_1066_MOESM1_ESM.docx]

**Table 1**. Characteristics of the victims (age, sex, psychiatric history), setting and circumstances of death, autopsy and histological findings, toxicological findings and analytical methods.

| **Reference** | **Country** | **Sex/Age** | **Psychiatric history** | **Setting and circumstances of death** | **Autopsy findings** | **Histological findings** | **Met-Hb %** | **NO2**  **value** | **NO_3_**  **value** | **Other toxicological findings** | **Analytical method** |
| --- | --- | --- | --- | --- | --- | --- | --- | --- | --- | --- | --- |
| ***Harvey et al.,* 2010 [41]** | New Zealand | M, 76 | Depressive syndrome;  P.S.A. | E.D. | N.A. | N.A.  . | A.M. 82.6% | NaNO2 in GC | N.A. | N.A. | N.A. |
| ***Mudan et al.*, 2020 [24]** | U.S.A. | M, 27 | N.A. | E.D. | N.A. | N.A. | A.M. >32.4% | N.A. | N.A. | N.A. | N.A. |
|  |  | F, 16 | N.A. | E.D.;  NaNO2 purchased online | N.A. | N.A. | A.M. >30% | N.A. | N.A. | N.A. | N.A. |
|  |  | M, 25 | N.A. | E.D. | N.A. | N.A. | P.M. 29% | N.A. | N.A. | N.A. | N.A. |
| **Durao et al., 2020 [26]** | Portugal | M, 37 | Depressive syndrome; Schizophrenia; P.S.A. | Outdoor (car); Suicide kit ordered on the internet | **Hypostasis**: brown,grey,blue,red  **Cyanosis**: extremities  **Other findings**: general signs of asphyxia | Pulmonary edema;  Coronary artery disease | N.A. | GC 16000 mcg/mL  GC 24000 mcg/mL NaNO2  B 0.03 mcg/mL | N.A. | Citalopram; Trazodone | Spectrophotometry for NaNO2 |
| ***Neth et al*., 2021 [25]** | U.S.A. | F, 17 | Bipolar disorder; substance use disorder;  P.S.A. | On route to the E.D.;  NaNO2 purchased online | N.P. | N.P. | A.M. 48% | N.A. | N.A. | N.A. | N.A. |
| ***Dean et al.,* 2021 [34]** | U.S.A. | F, 24 | No history of mental issues | Indoor | **Hypostasis**: Mottled purple-grey  **Other findings**: deep red-purple blood with no clots | N.A. | P.M. 62% | N.A. | N.A. | N.D. | Spectrophotometry for Met-Hb |
|  |  | M, 22 | No history of mental issues | Indoor;  Suicide notes | **Hypostasis**: blue-grey discoloration of the skin and hand nails  **Dark discolouration and increased fluidity of the blood** | Linear hemorrhages in the stomach and intestine mucosa | P.M. 33% | VH 3402,37 mcg/mL Na | N.A. | N.D. | Spectrophotometry for Met-Hb |
|  |  | M, 39 | [Personality Disorder](https://my.clevelandclinic.org/health/diseases/9784-paranoid-personality-disorder);  P.S.A. | Indoor | **Hypostasis**: red-purple and grey  **Cyanosis**: hand nails  **Dark discolouration and increased fluidity of the blood** | hemorrhagic gastritis;  Visceral congestion | P.M. 44% | N.D. | N.A. | N.D. | Spectrophotometry for Met-Hb |
| ***Durao et al.,* 2021 [38]** | Portugal | F, 37 | No history of mental issues | Indoor (bedroom); Suicide note | **Hypostasis**: greyish  **Other findings**: general signs of asphyxia | Myocardial changes;  Pulmonary edema | N.A. | HB 35mcg/mL | N.A. | Diazepam; Codeine; Quetiapine; Tramadol | Griess method |
| ***Barranco et al*., 2021 [29]** | Italy | M, 31 | Depressive syndrome | Outdoor (car) | **Hypostasis**: blue-red  **Cyanosis**: hands and feet | Pulmonary edema and intra-alveolar hemorrhages;  Myocardial changes | P.M. 73% | N.A. | N.A. | N.A. | UPLC-MS/MS  (Ultrahigh-performance liquid chromatography–tandem mass spectrometry technique) |
| ***Hwang et al*., 2021 [20]** | Korea | M, 28 | N.A. | Indoor (Home); Logged-in on suicide forum | **Hypostasis**: reddish-purple  **Cyanosis**: hands and feet  **Petechiae**: hand nails  **Other findings**: dark brown color of the face | Visceral congestion;  Pulmonary edema | P.M. 33% | GC 11220.1 mcg/mL  PF 181.0 mcg/mL | CSF 50.5 mcg/mL  GC 137.8 mcg/mL  PB 220.0 mcg/mL  HB 218.5 mcg/mL  PF 91.7 mcg/mL | N.A. | Blood gas analysis  (Met-Hb)  Ion chromatography  (nitrites/nitrates/  sodium nitrite) |
| ***Hickey et al.,* 2021 [19]** | Canada | 21 M; 7 F  32.8 (SD ± 18.84)  aa  Min. 17  Max. 86 | N.A. | N.A. | **Hypostasis**  N.A. (1 case);  Unremarkable (7 cases);  Different shades of purple (11 cases)  Different shades of grey (8 cases)  **Dark discolouration of the blood** (1 case) | N.A. | P.M. Met-Hb range 6%-92%  Test failed (2 cases)  N.A. (3 cases) | N.A. | N.A. | N.A. (3 cases)  Negative (9 cases)  Antihistamines (10 cases)  Antidepressants (6 cases)  Antiemetics (1 case)  Sub. of abuse (2 cases)  Others (4 cases) | N.A. |
| ***Huntington et al.,* 2021 [39]** | U.K. | F, 28 | N.A. | E.D. | N.A. | N.A. | A.M. 81% | N.A. | N.A. | N.A. | N.A. |
| ***Tomsia et al.,* 2021 [31]** | Poland | M, 23 | N.A. | Indoor ( bathroom) | **Hypostasis**: dark purple discoloration of the lips  **Cyanosis**: hands and fingernails | N.A. | N.D. | B 0.2 mcg/mL  U 24.6 mcg/mL  GC 220 mcg/mL  VH 57.7 mcg/mL  Costal cartilage 3.4 mcg/mL  Kidney 3.6 mcg/mL | N.A. | Negative | Griess method |
| ***Taus et al.,* 2021 [33]** | Italy | M, 28 | Depressive syndrome (mild) | Indoor | **Hypostasis**: brown-red  **Cyanosis**: Lips; finger nails | Pulmonary edema;  Visceral congestion | N.A: | traces on blood | B 403.031 mcg/mL | PB Et-OH | Capillary electrophoresis operating in the CIA mode coupled with UV detection |
|  |  | M, 33 | Depressive syndrome (severe) | Indoor (Living room) | **Hypostasis**: grey-blue-red  **Cyanosis**: extremities | N.A. | N.A. | N.D. | B 272.82 mcg/mL | Delta-THC | + |
| ***Bugelli et al.,* 2022 [30]** | Italy | M, 51 | No history of mental issues | Indoor (Bedroom); Suicide note | **Hypostasis**: greyish  **Cyanosis**: Hands (Subungueal)  **Dark discolouration of the blood**  **Other findings**: pulmonary edema; visceral congestion | Myocardial changes; Pulmonary edema and emphysema | P.M. >30% | N.D | PB 460 mcg/mL  U 48.3 mcg/mL | Alprazolam;  Et-OH | HPLC coupled to  High-Resolution Mass Spectrometry (HRMS); LC-MS  (Blood gas analysis using GEM Premier 5000 (Werfen) to  detect Met-Hb) |
|  |  | M, 35 | Anxious-depressive syndrome | Indoor (Bathroom) | **Hypostasis**: greyish and purple  **Cyanosis**: Hand nails  **Dark discolouration of the blood**  **Other findings**: pulmonary edema; visceral congestion | Myocardial changes;  Pulmonary emphysema and intra-alveolar haemorrhages | N.A. | N.D. | PB 280 mcg/mL  U 269 mcg/mL | Mirtazapine | + |
|  |  | F, 44 | No history of mental issues | Indoor;  Suicide note | **Hypostasis**: purple-bluish  **Cyanosis**: Lips and hand nails | Visceral congestion;  pulmonary emphysema;  brain edema;  Myocardial changes | P.M. >30% | N.D. | PB 378 mcg/mL  U <10 mcg/mL | Metoclopramide | + |
|  |  | M, 27 | No history of mental issues | Outdoor (mountainous area); Called suicide hotline declaring he was going to commit suicide | **Hypostasis**: blue-greyish  **Cyanosis**: Lips and hand nails | Visceral congestion;  Pulmonary emphysema | P.M. >30% | N.D. | PB 311 mcg/mL  U 102 mcg/mL | Metoclopramide | + |
| ***Wettstein et al.,* 2022 [36]** | U.S.A. | M, 25 | No history of mental issues | E.D. | **Other findings**: brown discoloration of the muscle tissue | N.A. | A.M. 28.8% | B Nitrate + Nitrite 5300 micromol/L | B Nitrate + Nitrite 5300 micromol/L | N.A. | N.A. |
| ***Mun et al.,* 2022 [21]** | Korea | M, 26 | N.A. | E.D. | N.A. | N.A. | A.M. 90.3% | N.A. | N.A. | N.A. | N.A. |
| ***Stephenson et al.,* 2022 [18]** | Australia | M, 74 | Depressive syndrome;  P.S.A. | N.A.;  Informed friends about his intentions to commit suicide | **Hypostasis**: blue-grey  **Dark discolouration of the blood**  **Other findings**: pulmonary edema | N.A. | N.A. | N.A. | N.A. | Diazepam;  Venlafaxine  Amlodipine;  Paracetamol; ​​Prochlorperazine | N.A. |
|  |  | M, 60 | Depressive syndrome | N.A.;  Suicide note | **Hypostasis**: blue-grey  **Dark discolouration of the blood**  **Other findings**: pulmonary edema | N.A. | P.M. 87.5%  CO-Hb 31% | N.A. | N.A. | Venlafaxine; Desvenlafaxine; Paracetamol; Buprenorphine; Metformin;  Mirtazapine | N.A. |
|  |  | M, 74 | Anxious-depressive syndrome; | N.A.;  Suicide note | **Hypostasis**: blue-grey  **Dark discolouration of the blood**  **Other findings**: pulmonary edema | N.A. | CO-Hb 51%  Met-Hb outside analytical limit | Urine dipstick positive | N.A. | Lorazepam;  Fentanyl; Metoclopramide; Pregabalin | N.A. |
|  |  | M, 69 | Depressive syndrome | N.A.;  Suicide note | **Hypostasis**: blue-grey  **Dark discolouration of the blood**  **Other findings**: pulmonary congestion | N.A. | Sample not suitable | Urine dipstick positive | N.A. | Sertraline | N.A. |
|  |  | F, 29 | Depressive syndrome;  P.S.A. | N.A.;  Farewell messages on the phone;  Suicide note | **Hypostasis**: blue-grey  **Dark discolouration of the blood**  **Other findings**: pulmonary congestion and edema | N.A. | CO-Hb 25% | Urine dipstick positive | N.A. | Naloxone | N.A. |
|  |  | M, 64 | No history of mental issues | N.A. | **Hypostasis**: blue-grey  **Dark discolouration of the blood**  **Other findings**: pulmonary edema | N.A. | Met-Hb N.D. | Urine dipstick positive | N.A. | Paracetamol;  Codeine;  Prochlorperazine; Ranitidine;  Loperamide | N.A. |
|  |  | F, 23 | Personality disorder; | N.A.;  Suicide note;  Internet search about suicide methods | **Hypostasis**: blue-grey  **Dark discolouration of the blood**  **Other findings**: pulmonary edema and congestion | N.A. | CO-Hb 51%  Met-Hb N.D. | Urine dipstick positive | N.A. | Metoclopramide; Quetiapine;  Paracetamol | N.A. |
|  |  | M, 64 | Depressive syndrome | N.A.;  Suicide notes | **Hypostasis**: blue-grey  **Dark discolouration of the blood**  **Other findings**: pulmonary edema and congestion | N.A. | CO-Hb 40%  Met-Hb N.D.  AM | Urine dipstick positive | N.A. | Et-OH;  Metoclopramide; Paracetamol;  Zolpidem | N.A. |
|  |  | M, 22 | N.A. | N.A.;  Suicide notes | **Hypostasis**: blue-grey  **Dark discolouration of the blood** | N.A. | Met-Hb N.D. | Urine dipstick positive | N.A. | Metoclopramide; Ranitidine;  Paracetamol | N.A. |
|  |  | M, 40 | N.A. | N.A.;  Suicide notes | **Other findings**: Advanced putrefactive changes | N.A. | Met-Hb N.D. | Urine dipstick positive | N.A. | Paracetamol; Metoclopramide;  Et-OH | N.A. |
| ***Loiseau et al.,* 2023 [22]** | France | F, 33 | Spectrum autism disorder | Indoor (Bedroom) | **Hypostasis**: purplish  **Cyanosis**: Lips and ears  **Petechiae**: inner surface of the scalp  **Other findings**: Visceral congestion; whitish foam in the tracheobronchial tree | Visceral congestion; intra-alveolar hemorrhages | N.A. | GC 30.9 mcg/mL | N.A. | Metoclopramide | RAMAN spectrometry;  Spectrophotometric study using the Saltzman's reagent for Gastrc Content analysis |
| ***Szórádová et al.,* 2023 [28]** | Slovak Republic | F, 19 | No history of mental issues | Outdoor (Vehicle) | **Hypostasis**: grey-purple; grey-brown  **Petechiae**: subpleural effusions  **Dark discolouration and increased fluidity of the blood**  **Other findings**: Visceral congestion | N.A. | P.M. >70% | N.A. | N.A. | N.A. | N.A. |
|  |  | M, 24 | Personality disorder;  P.S.A. | Outdoor (Cornfield) | **Hypostasis**: grey-blue; grey-brown  **Dark discolouration and increased fluidity of the blood**  **Other findings**: Visceral congestion | N.A. | P.M. 20.24% | Detected in GC and U, but value not specified | Detected in B, GC and U, but value not specified | Acetylsalicylic acid; Ibuprofen metabolite | Isotachophoresis with a conductivity detector. |
|  |  | F, 33 | Depressive syndrome | Indoor (Home) | **Hypostasis**: grey-blue-purple  **Petechiae**: subpleural effusions  **Dark discolouration and increased fluidity of the blood**  **Other findings**: Visceral congestion | N.A. | P.M. 54.5% | 5.51 mcg/mL | 219,72 mcg/mL | Metoclopramide | N.A. |
|  |  | M, 21 | Personality disorder | On route to the E.D. | **Hypostasis**: grey-purple  **Petechiae**: subpleural effusions  **Dark discolouration and increased fluidity of the blood**  **Other findings**: Old scars on both forearms; Visceral congestion | N.A. | P.M. 71.4% | N.A. | N.A. | N.A. | N.A. |
| ***Andelhofs et al.,* 2023 [32]** | Belgium | F, 18 | Mental health issues;  P.S.A. | Indoor (Bedroom) | **Hypostasis**: grey-brownish  **Cyanosis**: Lips and fingernails  **Other findings**: Healing self-harm injuries on the forearms | Pulmonary edema and intra-alveolar hemorrhages;  Visceral congestion;  Liver steatosis | P.M. 35% | Serum 38 mcg/mL  GC 1150 mcg/mL | N.A. | Negative | Spectrophotometric analysis;  Griess reaction; Macherey–Nagel. |
| ***Hikin et al.,* 2023 [1]** | U.K. | F 8; M 12  31.3 (SD ± 11.5)  Min. 14  Max. 49 | P.S.A (10 cases)  Depressive syndrome (4 cases)  Personality disorders (2 cases)  Other mental health issues (5 cases)  Eating disorder (1 case)  N.A. (4 cases)  Cases with > 1 condition (1) | Indoor (Bedroom 6 cases, Home 1 case, Living room 2 cases)  on route to E.D. (1 case)  E.D. (1 case)  N.A. (9 cases)  Suicide pact (2 cases) | N.A. | N.A. | N.A. | Blood range 0.107 – 372.64 mcg/mL  B ND (1 case) | Blood range 0.899mcg/mL – 1308 mcg/mL  B traces (1 case)  B N.D. (1 case) | Et-OH or illicit drugs (13 cases)  Paracetamol (9 cases)  Antiemetics (6 cases)  Antidepressants (4 cases)  Antihistamines (2 cases)  Antipsychotics (1 case)  Anticonvulsants (1 case)  N.D. (2 cases)  Others (4 cases) | Gas-phase chemiluminescence |
| ***Zhang et al.,* 2023 [23]** | U.S.A. | F, 19 | Depressive syndrome; post-traumatic stress disorder; ADHD; Borderline personality disorder | Indoor (Bedroom) | N.A. | N.A. | Sample unsuitable | U negative  Serum positive  VH positive | N.A: | N.A. | Mquant Nitrite Test Strip with Griess method |
|  |  | F, 43 | Anxious-depressive syndrome;  P.S.A. | Indoor (Hotel room) | N.A. | N.A. | Sample unsuitable | U negative  VH positive | N.A. | N.A. | Mquant Nitrite Test Strip with Griess method |
|  |  | F, 61 | Depressive syndrome (not diagnosed) | Indoor (Hotel room) | N.A. | N.A. | Sample unsuitable | U positive  VH positive | N.A. | N.A. | Mquant Nitrite Test Strip with Griess method |
|  |  | F, 16 | Anxious-depressive syndrome;  P.S.A. | E.D. | N.A. | N.A. | A.M. 8.5% | U positive  VH positive  Serum positive | N.A. | N.A. | Mquant Nitrite Test Strip with Griess method |
|  |  | F, 41 | Suicidal thoughts | On route to the E.D | N.A. | N.A. | A.M. 58% | Serum positive  VH positive | N.A. | N.A. | Mquant Nitrite Test Strip with Griess method |
| ***Zerbo et al*., 2023 [27]** | Italy | F, 20 | Eating disorder (Anorexia) | Indoor (Bedroom); Handwritten note on how to consume NaNO2 along with the website | **Hypostasis**: greyish-purple  **Cyanosis**: Lips  **Petechiae**: subpleural; laryngeal; glottal; tracheal mucosa  **Other findings**: foamy liquid in the tracheal lumen and at lung section | N.P. | P.M. 12.8% | N.A. | N.A. | Negative | AVOXimeter  4000 ITC oximeter on central blood to detect Met-Hb |
| ***Ječmenica et al.;* 2024 [35]** | Serbia | F, 47 | Depressive syndrome | Indoor (Home) | **Hypostasis**: grey-brownish;  **Other findings**: foamy liquid in the tracheal lumen; lung congestion | N.P. | P.M. 60.3% | PB 159.8 mcg/mL | PB 1597.9 mcg/mL | Metoclopramide;  Risperidone | Griess spectrophotometry (NO_2_)  UV spectrophotometry (NO_3_)  Evelyn-Malloy’s spectrophotometry (Met-Hb) |
| ***Kaubrytė et al.;* 2025 [40]** | Lithuania | F, 19 | N.A. | Indoor (Home); Suicide note | **Hypostasis**: cherry-brownish;  **Petechiae**: brain  **Other findings**: visceral congestion; pulmonary edema with foamy liquid;  **Dark discolouration of the blood** | N.P. | N.P. | GC positive | N.P. | Et-OH;  Amphetamines;  Atropine;  Quetiapine | N.A. |

Notes: P.S.A. Previous suicide attempt; HB (Heart blood); PB (Peripheral blood); U (Urine); VH (Vitreous Humor); GC (Gastric content); PF (Pericardial fluid); CSF (Cerebrospinal fluid); N.D. (Not detectable); N.A. (Not available); N.P. (Not performed)
